# Emergence of heteroresistance to carbapenems in Gram-negative clinical isolates from two Egyptian hospitals

**DOI:** 10.1186/s12866-024-03417-y

**Published:** 2024-07-26

**Authors:** Alaa G. Al-Shebiny, Riham M. Shawky, Mohamed Emara

**Affiliations:** Faculty of Pharmacy, Department of Microbiology and Immunology, Helwan University, P. O. Box: 11795, Ain-Helwan, Cairo, 01060564729 Egypt

**Keywords:** Heteroresistance, Subpopulations, Treatment failure, Carbapenems, Phenotype, Genotype

## Abstract

**Background:**

Antimicrobial resistance is a global concern, linking bacterial genotype and phenotype. However, variability in antibiotic susceptibility within bacterial populations can lead to misclassification. Heteroresistance exemplifies this, where isolates have subpopulations less susceptible than the main population. This study explores heteroresistance in Gram-negative bacteria, distinguishing between carbapenem-sensitive isolates and stable heteroresistant isolates (SHIs).

**Methods:**

A total of 151 Gram-negative clinical isolates including *Klebsiella pneumoniae*,* Pseudomonas aeruginosa*,* Escherichia coli*,* Acinetobacter baumannii* and *Proteus mirabilis* from various sources were included. Heteroresistant isolates and their stability were detected by disc-diffusion technique while genotypic analysis was carried out by PCR and efflux activity was assessed by ethidium bromide (EtBr)-agar cartwheel method.

**Results:**

A total of 51 heteroresistant subpopulations were detected, producing 16 SHIs upon stability-detection. Amplified resistance genes and EtBr-agar cartwheel method showed a significant difference between resistant subpopulations and their corresponding-sensitive main populations.

**Conclusion:**

Genotypic analysis confirmed that genetic mutation can lead to resistance development although the main populations were sensitive, thereby leading to treatment failure. This is a neglected issue which should be highly considered for better treatment outcomes.

**Supplementary Information:**

The online version contains supplementary material available at 10.1186/s12866-024-03417-y.

## Background

It is imperative to have a deeper comprehension of the emergence and dissemination of drug-resistant bacteria in order to address the issue of antibiotic resistance. In addition to better diagnostic instruments, new therapeutic approaches can guide the clinicians to select the most appropriate antimicrobial therapies. Heteroresistance (HR) phenomenon, in which a bacterial isolate contains subpopulations with reduced antibiotic susceptibility compared to the main population [1], can lead to treatment failure [2].

The clinical manifestation of polymicrobial illnesses may be further complicated by highly resistant subpopulations of heteroresistant bacteria [3]. One approach to frame the concept of HR is as an intermediate point between antibiotic sensitivity and resistance [4, 5].

There are two main mechanisms underlying HR as a phenomenon, the first is called polyclonal HR, in which a bacterial infection is followed by a subsequent infection with distinct bacterial isolate [1] as has been described for *Mycobacterium tuberculosis* and *Helicobacter pylori* [6, 7]. Rare spontaneous resistance mutants that are proportionately growing in the population under antimicrobial therapy can generate polyclonal HR [1]. Second, there is monoclonal HR which is generated from a single clone that differentiates into two populations (susceptible and resistant) in such case [1]. Genetic HR and physiological (non-genetic) HR are the two potential drivers of monoclonal HR. The primary source of monoclonal heterogeneity is genetic HR [1], which can also lead to point mutations, minor deletions, or tandem gene amplification [8], small deletions or point mutations [9], that lead to instability of such heterogeneous response. Therefore, a significant primary classification would be necessary to differentiate between the two possibilities [7]. It is also to be emphasized that the term “heteroresistance” has been used to describe mixed populations of bacteria with stable genetic differences, including closely related bacteria that formed co-infections with two different strains [10] or mutations [9, 11]. It is important to distinguish HR from other forms of subpopulations-mediated resistance such as persistence and tolerance [2, 12]. It is crucial to identify if subpopulations mediate resistance through tolerance, persistence, heterogeneity, or some other mechanism [4].

The clinical concern of HR detection arises from the possibility that treatment failure may occur due to the emergence of resistant subpopulations after antibiotic exposure [2]. Failure to detect HR by usual diagnostic testing, results in misclassification of some strains as susceptible [1]. In actuality, even in cases where a bacterial isolate is determined to be susceptible to a particular antibiotic, the likelihood of an antibiotic therapy successfully starts at 90% and decreases [13]. Consequently, there is a heavy cost associated with such unexplained treatment failure, and HR may be a contributing factor.

By contrast, no correlation between HR and treatment failure was defined in some studies [14] and cohort studies of vancomycin-treated heteroresistant subpopulations of vancomycin-susceptible *Staphylococcus aureus* (hVISA) [3, 15, 16]. So, to completely understand which HR-related factors in particular, the frequencies of heteroresistant subpopulations and their resistance levels are likely to alter the treatment outcome, further studies are required. Furthermore, at least some of the disparities between independent investigations may be explained by differences in sample sizes and detection techniques [4].

The main objective of this study was to explore the heteroresistance phenomenon in some Gram-negative isolates (GNIs) and shed the light and further characterize the difference between the carbapenems-sensitive and the heteroresistant GNIs on both phenotypic and genotypic levels.

## Materials and methods

Bacterial strains consecutively, non-duplicate isolated, *Klebsiella pneumoniae* (*n* = 60), *Pseudomonas aeruginosa* (*n* = 60), *Escherichia coli* (*n* = 16), *Acinetobacter baumannii* (*n* = 9) and *Proteus mirabilis* (*n* = 6).

A total of 151 different clinical GNIs were collected from the clinical pathology and pathophysiology lab in Kasr Al-Ainy Hospital and the National Cancer Institute (NCI), Cairo, Egypt, over a period of time from May 2020 to June 2021. Clinical isolates were recovered from various sources, including urine (*n* = 39), skin and skin structures (*n* = 35), blood (*n* = 22), respiratory samples (*n* = 19), pus (*n* = 18) and other infection sites (*n* = 18), using proper sampling techniques [16] from inpatients and outpatients units. The clinical isolates were purified using selective media where EMB agar and MacConkey agar were used alternatively for isolation of *E. coli*,* A. baumannii*, *K. pneumoniae* and *P. mirabilis* without prejudice, while *P. aeruginosa* was isolated on cetrimide agar for more specificity [17, 18]. All clinical isolates that had been purified were kept in an ultra-deep freezer (ilShin Europe B.V.) at -80 ºC as glycerol stocks for subsequent studies.

### Bacterial isolates identification

Apart from the process of cultivating on selective media noticing the form, color, and appearance of the colonies, pure clinical isolates were identified using biochemical testing subsequent to Gram staining [19]. Urea agar, citrate, lysine iron agar, motility indole ornithine and triple sugar iron media were used for this purpose [20]. All aforementioned tests were performed in Kasr Al-Ainy University Hospitals and NCI, Cairo, Egypt.

### Antimicrobial susceptibility testing

All clinical isolates were examined to determine whether it was susceptible to any particular class of antibacterial drug, such as cephalosporins (FEP, cefepime; FOX, cefoxitin), carbapenems (IPM, imipenem; ETP, ertapenem; MEM, meropenem; DOR, doripenem), fluoroquinolones (CIP, ciprofloxacin) aminoglycosides (CN, gentamicin), sulfonamides (SXT, trimethoprim/sulfametho-xazole), beta-lactam combination agents (AMC, amoxicillin/clavulanate), by disc diffusion method of Kirby Bauer on standard Mueller-Hinton agar (MHA) (Oxoid, England) [21].

The classification of isolates as susceptible, intermediate, or resistant was carried out as per the CLSI guidance 2020. For quality control during the entire process, three reference standard strains (*E. coli* ATCC 25922, *K. pneumoniae* ATCC 700603 and *P. aeruginosa* ATCC 27853) were recruited [22].

### Detection of heteroresistant subpopulations

Colonies that develop within an antibiotic disc’s zone of inhibition and impact the primary populations, the latter being interpreted as susceptible isolates, were identified as heteroresistant subpopulations [23]. One colony was picked up and purified using streak plate technique [24] and stored as a glycerol stock at -80 ºC for further investigations. The size and distance of the developed colony from the antibiotic disc were not taken into consideration during the random selection process.

### Heteroresistance stability detection

The resistance’s stability was examined by determining whether the pure clones isolated from the resistant subpopulations exhibited a reduced resistance phenotype after growing for 40–50 generations in the absence of antibiotics [25, 26]. All colonies, grown in the inhibition zone of a sensitive main population, were sub-cultured for 50 generations without antibiotic exposure.

Antimicrobial susceptibility testing (AST) was performed frequently against the relevant antibiotic every 5 sub-cultures to confirm HR stability. A resistant subpopulation is considered stable heteroresistant when the 10th AST (i.e. after the 50th sub-culturing), gives a resistant interpretation as per CLSI recommendations [9]. If the susceptibility in at least one of the cultures manifestly returned to that of the original parental isolate, the resistance was considered unstable [27].

### Phenotypic detection of carbapenemase genes

All stable heteroresistant isolates (SHIs), detected around carbapenems discs (i.e. six isolates), were tested for carbapenemase genes production using the Carbapenem Inactivation Method (CIM) according to CLSI M100 2020 [28].

### Detection of efflux pumps by cartwheel method

Efflux pumps activity was detected as previously described [29]. *E. coli* ATCC 25922 was used as a control. The plates were examined under a UV transilluminator. According to Patil and coworkers, the absence of fluorescence indicates the presence of a functioning efflux pump or pumps in the resistant subpopulations.

### Qualitative analysis of biofilm production

All stable heteroresistant strains were tested for biofilm production in an attempt to detect the difference between the heteroresistant and sensitive populations. Production of biofilm was studied by culturing the 16 SHIs on Congo Red Agar (CRA). Inoculated agar was incubated for 48 h at 37 °C and subsequently 2–4 days at room temperature. Biofilm-producing strains grow on CRA, form colonies that are partially or entirely black, while non-producing strains produce mucoid colonies that are somewhat reddish-white [30].

### Molecular detection of metallo-β-lactamase (MBL) genes by PCR

In the current work, all SHIs to carbapenems were chosen for a genotypic investigation of the HR phenomenon from species including *K. pneumoniae*,* E. coli* and *P. aeruginosa*.

GeneJET Genomic DNA Purification Kit was used for DNA extraction and purification from bacterial cells. Nano-drop quantification using Q3000-Quawell 0.5-5000 ng/µl was performed to standardize the DNA amount in each sample [31].

The selection of genes for amplification was based on their incidence in Egypt and prevalence in the examined microorganisms [32–35]. Seven genes namely, *bla*_IMP−1_, *bla*_VIM−2_, *bla*_NDM−1_, *bla*_OXA−48like_, *bla*_SIM−1_, *bla*_SPM−1_ and *bla*_GIM−1_, were amplified in the stable carbapenems-heteroresistant subpopulations and their corresponding sensitive main populations.

Primers that have been used to detect carbapenems-resistant genes in the heteroresistant isolates of interest were designed as previously described by Laurent and coworkers [36] confirmed and analyzed on Primer-BLAST website.

https://www.ncbi.nlm.nih.gov/tools/primer-blast/primertool.cgi.

Primers sequences are listed in Table 1.

**Table 1:** List of oligonucleotides used in this study

Name	Gene	Sequence (5’-3’)	Product Size (bp)	Reference
IMP-F	*bla*_IMP−1_	GGAATAGAGTGGCTTAAYTCTC	232	[36]
IMP-R		GGTTTAAYAAAACAACCACC		
SPM-F	*bla*_SPM−1_	AAAATCTGGGTACGCAAACG	271	
SPM-R		ACATTATCCGCTGGAACAGG		
VIM-F	*bla*_VIM−2_	GATGGTGTTTGGTCGCATA	390	
VIM-R		CGAATGCGCAGCACCAG		
GIM-F	*bla*_GIM−1_	TCGACACACCTTGGTCTGAA	477	
GIM-R		AACTTCCAACTTTGCCATGC		
SIM-F	*bla*_SIM−1_	TACAAGGGATTCGGCATCG	570	
SIM-R		TAATGGCCTGTTCCCATGTG		
OXA-F	*bla*_OXA−48−like_	GCGTGGTTAAGGATGAACAC	438	
OXA-R		CATCAAGTTCAACCCAACCG		
NDM-F	*bla*_NDM−1_	GGTTTGGCGATCTGGTTTTC	621	
NDM-R		CGGAATGGCTCATCACGATC		

The PCR mixture was carried out in a final volume of 50 µl comprising 5 µl of DNA template (5–20 ng/µl), 25 µl of PCR master mix, 2.5 µl of forward primer (0.1–0.5 µM), 2.5 ml of reverse primer (0.1–0.5 µM), and 15 µl nuclease-free water. The thermal cycler (MultiGene OptiMax ThermoCycler, Labnet) was programmed with the following conditions:-

#### Conventional PCR

initial denaturation at 95 ºC for 5 min, followed by 35 cycles of denaturation at 94 ºC for 30 s, annealing at 56 ºC, for *bla*_OXA−48_ and *bla*_NDM−1_, and 52 ºC, for *bla*_GIM−1_ and *bla*_VIM−2_, for 40 s, extension at 72 ºC for 30 s, and final cycle of amplification at 72 ºC for 10 min [37].

#### Gradient PCR

 was programmed for *bla*_IMP−1_, *bla*_SIM−1_ and *bla*_SPM−1_ starting from 52 ºC reaching 58 ºC in an attempt to reach the optimum annealing temperature for *bla*_IMP−1_, *bla*_SIM−1_ and *bla*_SPM−1_ genes using the touch up technique mentioned elsewhere [38]. Briefly, initial denaturation at 95 ºC for 5 min, followed by 35 cycles of denaturation at 94 ºC for 30 s, annealing starting at 52º C till reach 58 ºC, increasing by 0.5 ºC each cycle, extension at 72º C for 30 s, and final cycle of amplification at 72 ºC for 10 min.

#### Touchdown PCR

was programmed for the genes which are not detected by gradient PCR; in order to increase sensitivity and specificity in amplification. The annealing temperature was designed starting from 60 ºC till 53 ºC. OligoCalc, an online tool for melting temperature (T_m_) calculation and oligonucleotide properties, was employed where it recommends basic, salt-adjusted and nearest-neighbor Tm estimations.

http://www.basic.northwestern.edu/biotools/oligocalc.html.

Lab positive and negative controls for each PCR were included. After agarose gel electrophoresis with EtBr, the PCR products were visualized under UV light.

## Results

### Identification of clinical isolates and antimicrobial susceptibility testing

A total of 151 GNIs, were identified by Gram staining and culturing methods. Additional confirmatory biochemical tests were carried to further identify the isolates. The susceptibility pattern of all collected isolates to tested antibiotics is shown below in Table 2.

**Table 2:** Antibiotic susceptibility pattern of the tested clinical isolates

Microorganisms	Most effective antibiotic/ %	Lowest effective antibiotic/ %
*K. pneumoniae*	IPM**/ 41.6%**	FEP and AMC**/ 6.7% each**
*P. aeruginosa*	IPM**/ 30%**	CIP**/ 8.33%**
*E. coli*	FOX**/ 62.5%**	DOR, FEP, AMC, ETP & SXT**/ 25%**
*A. baumannii*	IPM, DOR & CN**/ 22.22% each**	FEP, MEM & CIP**/ 0%**
*P. mirabilis*	ETP, DOR & IPM**/ 50% each**	CN, FOX, AMC & CIP**/ 0%**

Imipenem was the most effective antibiotic with a percent of 37.75% in contrast to amoxicillin-clavulanic acid, the less effective, with a percent of 9.7% (*p*-value > 0.05)

### Heteroresistant isolates and stability detection

Heteroresistant subpopulations were detected by the growth of colonies in the inhibition zone of each antibiotic disc [23]. Table 3 shows the susceptibility pattern of tested microorganisms against the used antibiotics, the outline of SHIs of total detected HR cases against all used antibiotics and the prevalence of HR in different microorganisms and its stability.

**Table 3:** Antibiotic susceptibility patterns of the clinical isolates

Antibacterial agent	No. of stable heteroresistant isolates (No. of Susceptible isolates) ^a^				
	K. pneumoniae	E. coli	*P*. aeruginosa	A. baumanii	*P*. mirabilis
CIP	2 (12)	1 (6)	1 (5)	0 (0)	0 (0)
MEM	1 (19)	1 (6)	1 (13)	0 (0)	0 (2)
IPM	1 (25)	0 (9)	0 (18)	0 (2)	0 (3)
DOR	0 (11)	0 (4)	1 (12)	0 (2)	0 (3)
ETP	1 (8)	0 (4)	ND	ND	0 (3)
CN	1 (24)	1 (7)	1 (15)	0 (2)	0 (0)
FOX	1 (13)	1 (10)	ND	ND	0 (0)
SXT	1 (8)	0 (4)	ND	0 (0)	0 (1)
FEP	0 (4)	0 (4)	0 (13)	0 (0)	0 (2)
AMC	0 (4)	1 (4)	ND	ND	0 (0)
SHI/ HI (Total sensitive isolates)	8/ 19 (128)	5/ 11 (58)	3/ 19 (25)	0/ 2 (6)	0/ 0 (14)

ND = not detected

^a^ Criteria as published by CLSI (2020) M100-ED30

HR has been detected in 27.5% and 30.4% of sensitive clinical isolates of meropenem and ciprofloxacin, respectively. (*P-*value is > 0.05)

*P. aeruginosa* has the highest level of HR incidence (25%), but *E. coli* has the highest HR stability percentage (45.5%). *Proteus mirabilis* recorded the lowest level (0%) followed by *K. pneumoniae* (14.8%) of HR incidence. *K. pneumoniae* has a prevalence of (42.1%) for HR stability. (*P-*value is > 0.05)

Repetitive susceptibility testing over 50 generations verified HR. Out of the 51 heteroresistant isolates that were found, only 16 isolates (or 31%) never reverted to susceptibility. Eleven out-patient isolates and five in-patient isolates made up the SHIs.

### Phenotypic characterization of efflux pumps activity, slime formation and carbapenemase genes production reflects the resistant nature of the subpopulations

**Fig. 1:**
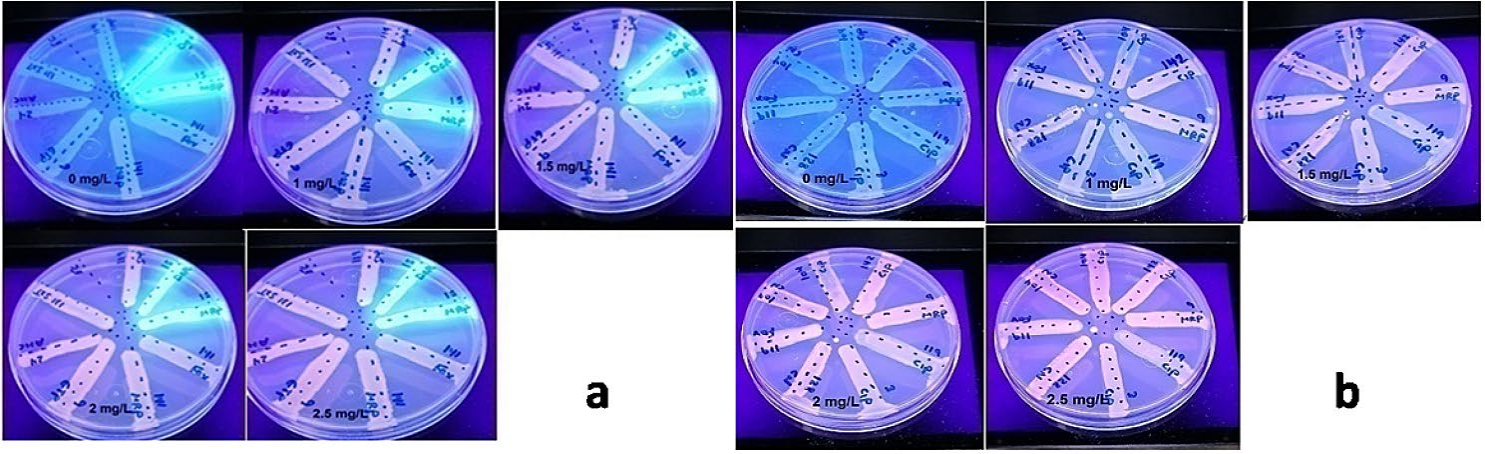
Efflux pumps activity detection in resistant subpopulations. **a**: for isolates; 119IPM, 15DOR, 15MRP, 141FOX, 141MEM, 9ETP, 24AMC and 131SXT. **b**: for isolates; 104CN, 104CIP, 142CIP, 9MEM, 119CIP, 3CIP, 128CN and 119FOX. Five isolates: 141FOX, 119CIP, 3CIP, 104CN and 104CIP reveal efflux pumps activity detected by absence of fluorescence upon examination by UV transilluminator (*p-*value > 0.05)

Out of 16 SHIs, five isolates (31.25%) revealed efflux pump activity upon detection by EtBr-agar cartwheel method (Fig. 1).

The fluorescent light of 15DOR and 15MRP even in 0 mg/L EtBr is due to pigments (pyoveridin and pyocyanin) producing fluorescence under UV light. It is considered as an obstacle to detect efflux pumps activity in *P. aeruginosa* using this method.

**Fig. 2:**
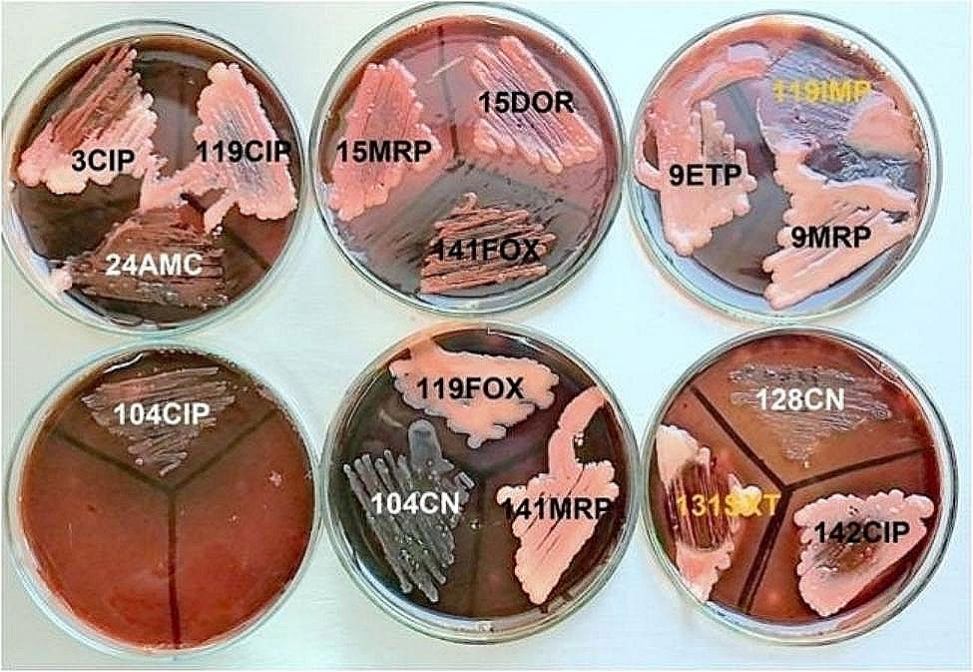
Biofilm formation detection in resistant subpopulations. Isolates, 104CIP, 104CN, 128CN and 24AMC exhibited biofilm formation (*p*-value > 0.05)

Testing biofilm formation in SHIs and their corresponding main populations revealed that none of the main populations were biofilm producers. Just four isolates (25%) of SHIs exhibited biofilm formation, as illustrated in Fig. 2.

Interestingly, all the tested carbapenems-SHIs (6 isolates) were found to be positive for carbapenemase production. This was confirmed by genotypic analysis which, revealed that all tested isolates produced at least one carbapenemase as illustrated in Table 4.

**Table 4:** Distribution of carbapenemase genes in stable carbapenems heteroresistant isolates

Stable HR isolates *N* (%)*	CIM *N*,% **	Carbapenemase genes *N* (%) **						
		bla_GIM-1_	bla_IMP-1_	bla_SPM-1_	bla_VIM-2_	bla_OXA-48 like_	bla_SIM-1_	bla_NDM-1_
***K. pneumoniae*** **3 (50%)**	3, 100%	0 (0%)	0 (0%)	0 (0%)	1 (33.3%)	1 (33.3%)	0 (0%)	3 (100%)
***E. coli*** **1 (16.67%)**	1, 100%	0 (0%)	0 (0%)	0 (0%)	0 (0%)	1 (100%)	0 (0%)	1 (100%)
***P. aeruginosa*** **2 (33.33%)**	2, 100%	2 (100%)	0 (0%)	0 (0%)	2 (100%)	0 (0%)	0 (0%)	1 (50%)
**Total = 6 (100%)**	6, 100%	2 (33.3%)	0 (0)%	0 (0)%	3(50%)	2 (33.3%)	0 (0)%	5 (83.3%)

* Percentages are calculated from the total six SHIs. ** Percentages are calculated from the total no. of stable resistant isolates in each species

Genes of *bla*_SPM−1_, *bla*_SIM−1_ and *bla*_IMP−1_ were not detected in any of the tested 6 SHIs. On the other hand and in contrast to their sensitive main population, *bla*_NDM−1_, *bla*_OXA−48 like_, *bla*_GIM−1_ and *bla*_VIM−2_ were detected in 5, 2, 2 and 3 out of 6 SHIs, respectively.

**Fig. 3:**
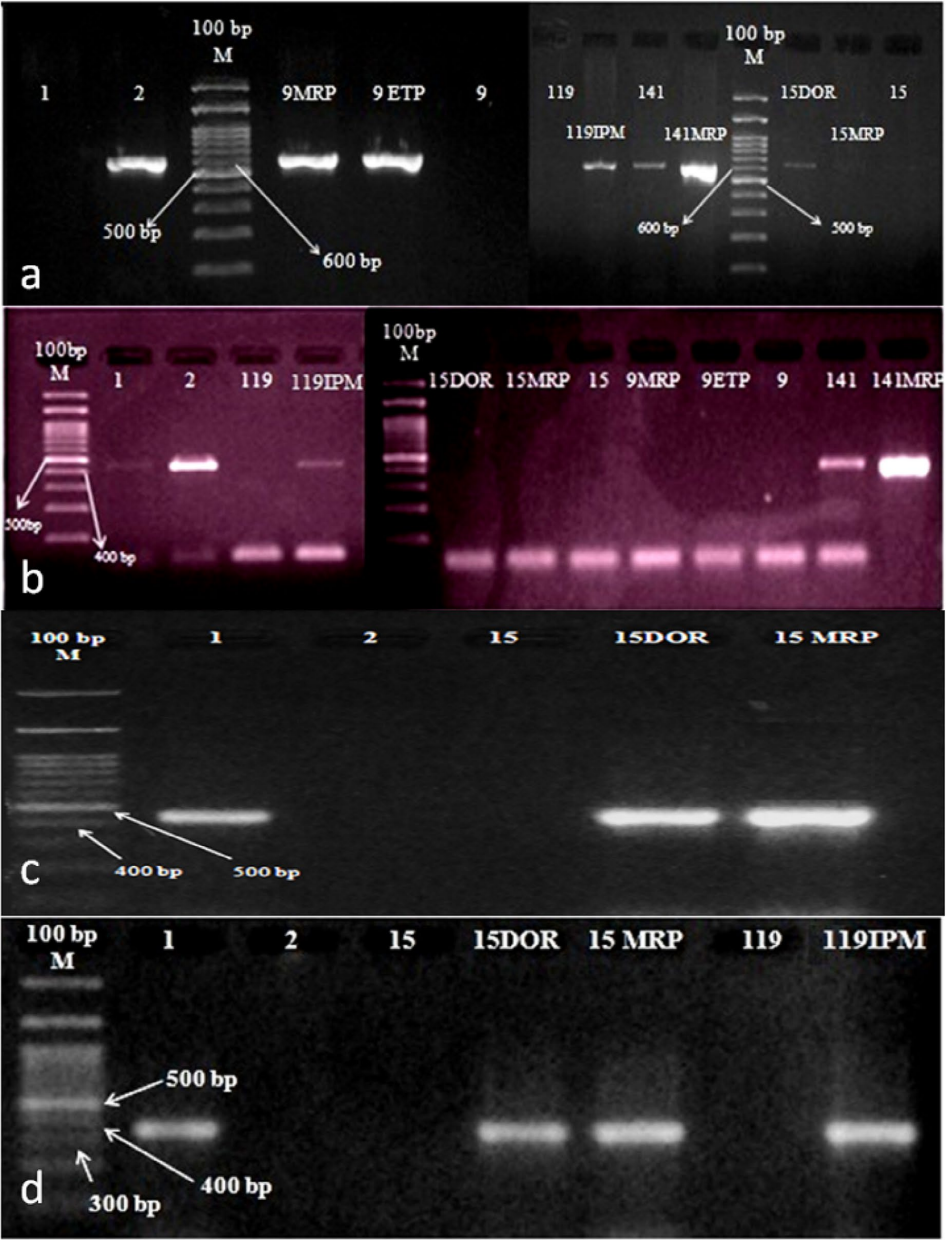
Carbapenemase genes amplification in resistant subpopulations and their corresponding sensitive main populations. **a**: Ethidium Bromide-stained gel shows PCR products of *bla*_NDM−1_ with size of 621 bp; lane 1, negative control; lane 2, positive control; M, 100 bp marker (ladder). (*p-*value > 0.05). **b**: Ethidium bromide-stained gel shows PCR products of *bla*_OXA−48 like_ with size of 438 bp; lane 1, negative control; lane 2, positive control; M, 100 bp marker (ladder). (*p-*value > 0.05). **c**: Ethidium bromide-stained gel shows PCR products of *bla*_GIM−1_ with size of 477 bp; lane 1, positive control; lane 2, negative control; M, 100 bp marker (ladder). (*p-* value < 0.05*). **d**: Ethidium bromide-stained gel shows PCR products of *bla*_VIM−2_ with size of 390 bp; lane 1, positive control; lane 2, negative control; M, 100 bp marker (ladder).(*p-*value > 0.05)

In (Fig. 3, A-D), sensitive main populations and the associated resistant subpopulations are compared.

Carbapenem resistance genes were identified across various infection types. The *bla*_VIM-2_ gene was detected in two samples obtained from wounds and in a third sample from urine. Conversely, all instances of *bla*_GIM-1_ were associated with wound infections. The *bla*_OXA-48 like_ gene was detected in only two samples: one from a urinary tract infection and another from wound pus. The *bla*_NDM-1_ gene was identified in samples from wounds, urine, and pus, as well as in two isolates obtained from blood cultures.

**Fig. 4:**
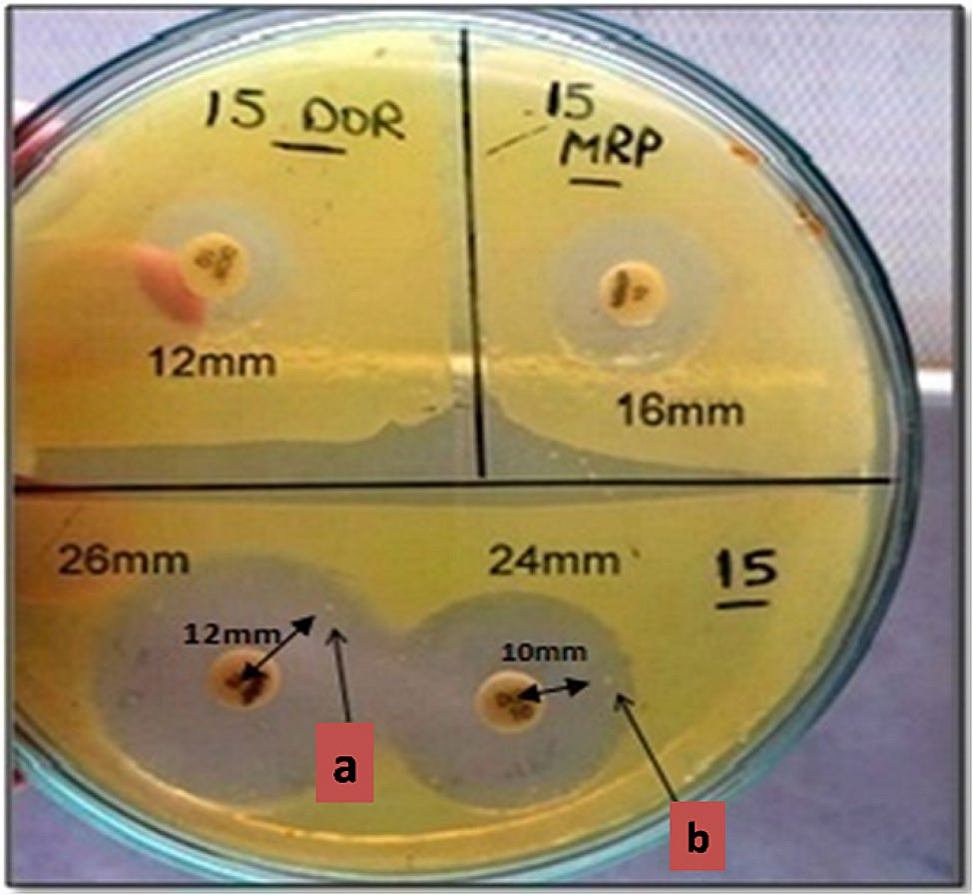
Resistance levels differ according to the Colonies’ position away from the disc center. **a**: a colony appeared around meropenem disc away from the disc centre by 12 mm. **b**: a colony appeared around doripenem disc away from the disc centre by 10 mm

Just as an observation, the closest colony to the antibiotic disc exhibits a narrower zone of inhibition compared to the one nearest to the inhibition zone edge of the same antibiotic for the same isolate. This observation was detected in isolate no.15 and its subpopulations; 15MRP and 15DOR (Fig. 4), in an attempt to clarify HR mechanism.

(Fig. 3A, C and D) indicates that 15DOR were triple positive for *bla*_NDM−1_, *bla*_GIM−1_ and *bla*_VIM−2_. On the other hand, *bla*_NDM−1_ couldn’t be detected in 15MRP, while *bla*_GIM−1_ and *bla*_VIM−2_ were detected. However, a closer examination of the gene expression levels using the Q-PCR technique is required to provide a more thorough explanation for this observation.

Table 5 presents an overview of the genotypic and phenotypic traits of SHIs in our investigation.

**Table 5:** Phenotypic and genotypic characteristics of stable heteroresistant subpopulations

Microorganisms		*P*. aeruginosa			K. pneumoniae								E. coli				
Isolates code		3CIP	15DOR	15MRP	9ETP	9MRP	119IPM	119FOX	119CIP	128CN	131SXT	142CIP	24AMC	104CIP	104CN	141MRP	141FOX
**Phenotypic characters**	**Efflux pumps activity (EtBr conc.)**	√ (2 mg/L)	X	X	X	X	X	X	√ (2 mg/L)	X	X	X	X	√ (2 mg/L)	√ (1.5 mg/L)	X	√ (2 mg/L)
	**Biofilm formation**	X	X	X	X	X	X	X	X	√	X	X	√	√	√	X	X
**Genotypic assessment** **(Carbapenemase genes)**	***bla***_**VIM−2**_	ND	√	√	X	X	√	ND	ND	ND	ND	ND	ND	ND	ND	X	ND
	***bla***_**SPM−1**_	ND	X	X	X	X	X	ND	ND	ND	ND	ND	ND	ND	ND	X	ND
	***bla***_**SIM−1**_	ND	X	X	X	X	X	ND	ND	ND	ND	ND	ND	ND	ND	X	ND
	***bla***_**GIM−1**_	ND	√	√	X	X	X	ND	ND	ND	ND	ND	ND	ND	ND	X	ND
	***bla***_**IMP−1**_	ND	X	X	X	X	X	ND	ND	ND	ND	ND	ND	ND	ND	X	ND
	***bla***_**NDM−1**_	ND	√	X	√	√	√	ND	ND	ND	ND	ND	ND	ND	ND	√	ND
	***bla***_**OXA−48**_	ND	X	X	X	X	√	ND	ND	ND	ND	ND	ND	ND	ND	√	ND

(√) and (X) symbols are used for positive and negative results of detection, respectively while (ND) means not detected

## Discussion

Although the term HR was initially used in the 1940s, some studies have looked at its genetic roots or how it may contribute to treatment failure [5]. The ongoing growth of HR and its complex regulations make its emergence deeply alarming [1]. This study comprised 151 distinct GNIs acquired from several different sources, comprising *Klebsiella pneumoniae*,* Pseudomonas aeruginosa*,* Escherichia coli*,* Acinetobacter baumannii*, and *Proteus mirabilis*. Out of 51 detected heteroresistant isolates (18% of 282 susceptible bacteria-antibiotic combinations), 31% of SHIs could be identified applying the disc-diffusion approach. Using PCR for genotypic analysis and the EtBr-agar cartwheel method for efflux activity assessment, significant differences were identified between heteroresistant isolates and their related main populations.

HR has been detected in different sample types against different antibiotics. Carbapenems are examples against which microorganisms exhibit heterogeneous response with different prevalence percentages in different studies ranging from 0% of different gram-negative clinical isolates in Sweden [9] to 100% in carbapenemases-producing *K. pneumoniae* in Greece [39]. Several studies revealed that HR occurs relatively often in *K. pneumoniae* clinical isolates [40]. In this study, *K. pneumoniae* was more prevalent (43.8%) among the 16 detected HR isolates followed by *E. coli* (31.3%). Additionally, unstable HR was widespread and detected in 69% of HR cases, yielding an overall estimated frequency of unstable HR of 12.48% (35 out of 282 bacteria–antibiotic combinations). Interestingly, for drugs where frequent mutations can cause resistance or where resistance genes can be amplified, the probability of observing HR might increase, such as in meropenem and ciprofloxacin drugs where HR has been detected in 27.5% and 30.4% of sensitive clinical isolates, respectively.

Several studies proved that HR is an unstable phenomenon where 100% [40], 88% [9] and 84.6% [41] of detected heteroresistant isolates were fully or partially reverted back to the level of susceptibility of the main population. In the current study, 51 heteroresistant subpopulations have been detected within 282 susceptible (out of 1234) bacteria–antibiotic combinations. Within this context, 35 isolates (68.63%) were shown to be unstable **(**Table 3**)**.

Mechanisms responsible for the surfacing of heteroresistant *K. pneumoniae* subpopulations have been previously identified as antibiotic-resistant factors in *Klebsiella* and other *Enterobacterials*. These mutations have often resulted in unstable HR including lipid-A modifications, over-expression of pump systems, or resistance genes amplification. In this study, different carbapenemases genes, namely, *bla*_NDM−1_, *bla*_VIM−2_, *bla*_GIM−1_, *bla*_SIM−1_, *bla*_SPM−1_, *bla*_IMP−1_ and *bla*_OXA−48 like_ were amplified. As mentioned earlier, *bla*_SPM−1_, *bla*_SIM−1_ and *bla*_IMP−1_ have not been detected in any of the six stable carbapenems-heteroresistant isolates. But the remaining genes were detected in carbapenems-resistant subpopulations by different ratios while nearly all these genes were absent in the corresponding main populations as shown in Table 4. It is assumed that these mutants survived the antibiotics effect and became superbugs by the activity of resistance genes.

Other HR mechanisms are down-regulation of porins and/or over-expression and activity of efflux pumps [42, 43]. These two mechanisms contributed to carbapenems-HR in *P. aeruginosa* [43, 44]. In this investigation, 31.25% of the SHIs were at least partially mediated by efflux pumps; this is in contrast to the corresponding main populations that did not exhibit any efflux pump activity. Those five isolates consist of three samples (119CIP, 104CIP and 104CN) obtained from urinary tract infections, from urine samples, another (141FOX) from a purulent sample (pus), and the fifth isolate (3CIP), from a respiratory tract infection obtained via sputum sample.

With regard to bacterial biofilm, little is known about its effect on HR, despite its heterogeneous response to antibiotics. Only one example described the presence of colistin-heteroresistant subpopulations in *K. pneumoniae* [45, 46]. In contrast to the aforementioned studies, it was demonstrated that biofilm formation in *K. pneumoniae* does not link with amikacin-HR [41]. Among a variety of phenotypic methods, the Congo Red Agar (CRA) method is a simple, cost-effective, sensitive, and specific method that can be used by clinical microbiology laboratories for screening of slime or slime-like substances [29].

Some studies reported that none of the *P. aeruginosa* strains tested showed positivity by CRA method suggesting that the CRA test may not be useful for identifying the exopolysaccharide layer-producing non-fermenting GNIs, especially *Pseudomonas* species [29].

In the existing study, slime production detection using CRA method discloses a biofilm formation nature in 4/16 (25%) SHIs, three of which (24AMC, 104CIP and 104CN) were obtained from urine samples and the forth one (128CN) accounts for a blood sample, **(**Fig. 2**)** while 12 SHIs (75%) exhibiting no biofilm production.

Preliminary data in which SHIs have been screened for susceptibility to β-lactam\β-lactamase inhibitor combinations (piperacillin\tazobactam and cefoperazone\sulbactam) as treatment alternatives resulting in major foremost resistance to the used combinations (data are not shown).

It is important to underline that the frequency of detected HR will depend on the detection method (for example, PAP test, E-tests, disk diffusion or broth microdilution) and the HR definition chosen (for example, the cut-off resistance level chosen to identify a resistant subpopulation) in addition to the selected clinical strains and the antibiotics tested [8]. For example, in our study we might have missed true HR cases if HR was not assumed following the first disc diffusion test. This makes comparisons of studies difficult and emphasizes the importance of approving a standard method and definition to identify HR in clinical isolates.

Moreover, two issues may compromise the rigorous procedure and the reproducibility of this research as well as data generalization. First, the small sample size may not be representative of the population, resulting in imprecise results and questioning the validity of the study and thereby can lead to fallacious conclusions and hence underpowered studies. Second, further molecular techniques such as sequencing analysis should have been employed to confirm PCR data. Nonetheless, the findings demonstrate that resistance genes amplification could contribute to HR incidence and should be taken into account when choosing an antibiotic. Improving detection techniques to find resistant cell subpopulations is a major clinical issue, so this is not trivial issue.

To conclude, heteroresistance (HR) poses significant challenges for microbiologists, healthcare providers, and patients. Diagnosis using established methods is often inaccurate, leading to treatment failures and potential development of drug-resistant bacteria. Improved techniques are urgently needed to detect HR accurately in pathogens. Studies so far, mainly in animal models or in-vitro, may not fully reflect host environments. Factors such as pathogen-host interactions and diverse subpopulations must be further explored through additional in-vivo studies. Whole genome sequencing holds promise for distinguishing SHIs from susceptible populations in future research.

### Electronic supplementary material

Below is the link to the electronic supplementary material.

Supplementary Material 1

## Data Availability

No datasets were generated or analysed during the current study.
