# Supplementary material for: Emergence of heteroresistance to carbapenems in Gram-negative clinical isolates from two Egyptian hospitals
Source: BMC Microbiol. 2024 Jul 26;24:278. doi: 10.1186/s12866-024-03417-y (PMC11282848; doi:10.1186/s12866-024-03417-y)
Supplement: Supplementary file 1 — Supplementary Material 1 [file 12866_2024_3417_MOESM1_ESM.docx]

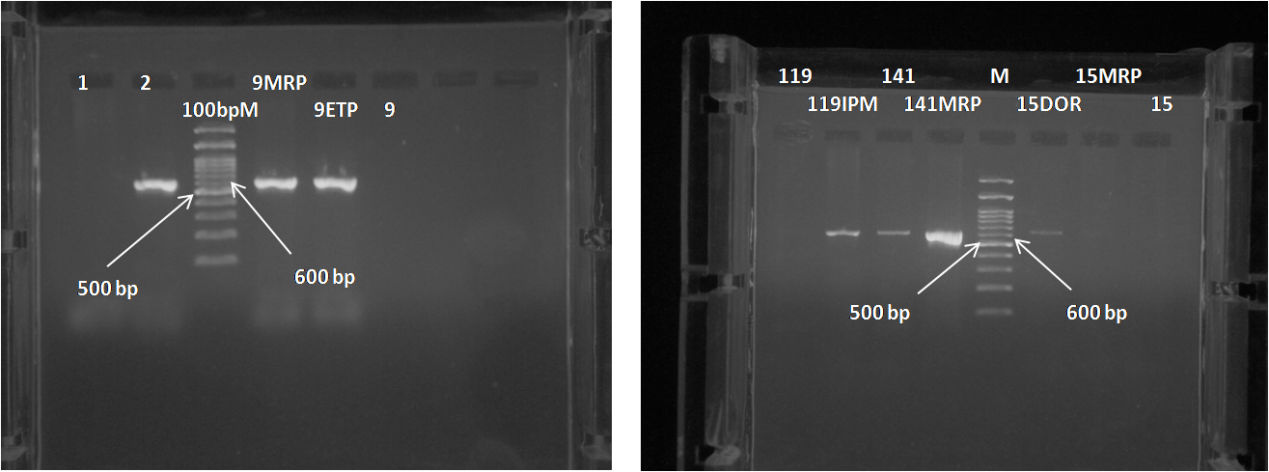


**Fig. 3 Carbapenemase genes amplification in resistant subpopulations and their corresponding sensitive main populations**

a: Ethidium Bromide-stained gel shows PCR products of *NDM* gene with size of 621 bp; lane 1, negative control; lane 2, positive control; M, 100 bp marker (ladder). (*p-*value >0.05).


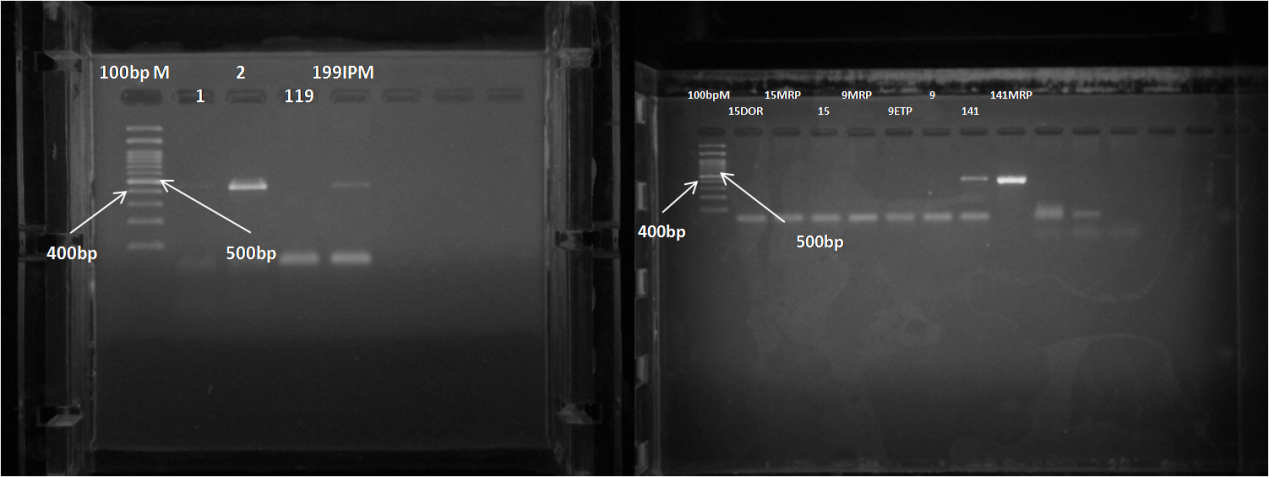


**Fig. 3 Carbapenemase genes amplification in resistant subpopulations and their corresponding sensitive main populations**

b: Ethidium bromide-stained gel shows PCR products of *OXA* gene with size of 438 bp; lane 1, negative control; lane 2, positive control; M, 100 bp marker (ladder). (*p-*value >0.05).


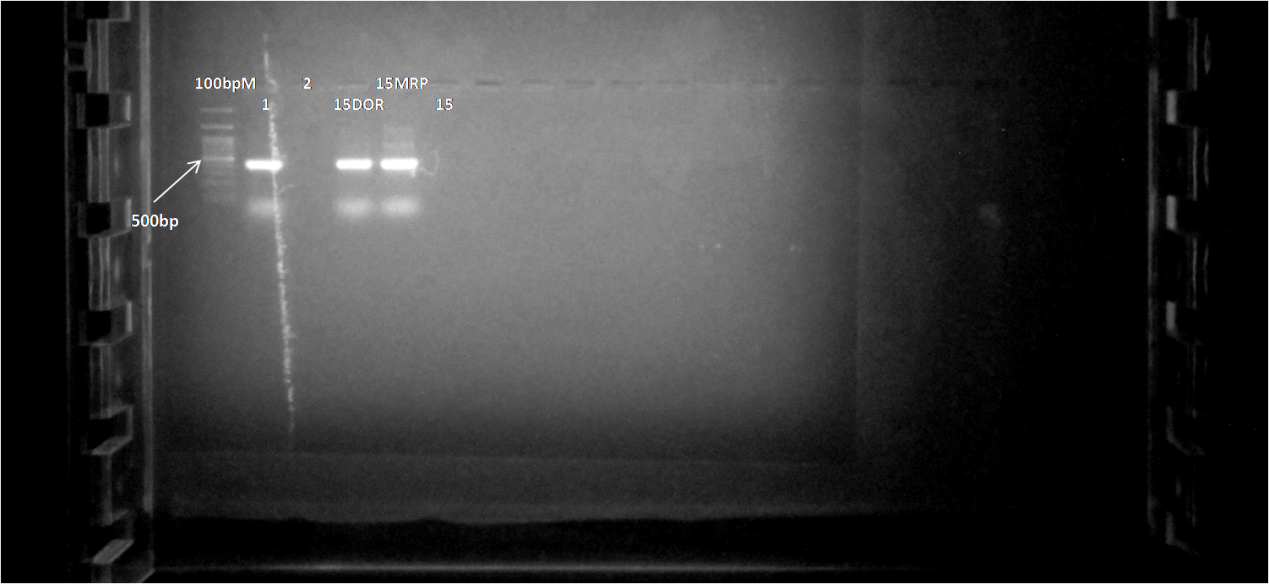


**Fig. 3 Carbapenemase genes amplification in resistant subpopulations and their corresponding sensitive main populations**

c: Ethidium bromide-stained gel shows PCR products of *GIM* gene with size of 477 bp; lane 1, positive control; lane 2, negative control; M, 100 bp marker (ladder). (*p-* value <0.05*).


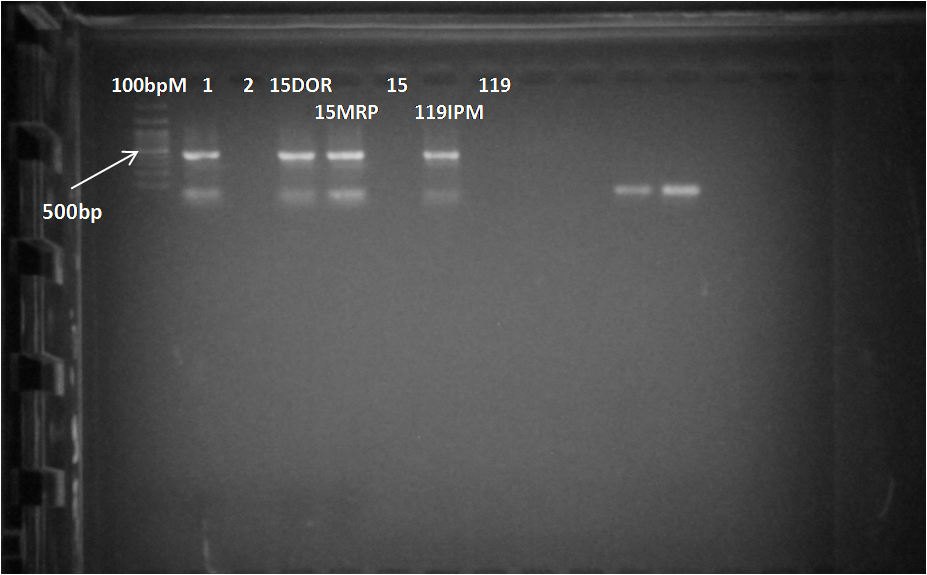


**Fig. 3 Carbapenemase genes amplification in resistant subpopulations and their corresponding sensitive main populations**

d: Ethidium bromide-stained gel shows PCR products of *VIM* gene with size of 390 bp; lane 1, positive control; lane 2, negative control; M, 100 bp marker (ladder). (*p-*value >0.05)
